# YOLOC-tiny: a generalized lightweight real-time detection model for multiripeness fruits of large non-green-ripe citrus in unstructured environments

**DOI:** 10.3389/fpls.2024.1415006

**Published:** 2024-07-05

**Authors:** Zuoliang Tang, Lijia Xu, Haoyang Li, Mingyou Chen, Xiaoshi Shi, Long Zhou, Yuchao Wang, Zhijun Wu, Yongpeng Zhao, Kun Ruan, Yong He, Wei Ma, Ning Yang, Lufeng Luo, Yunqiao Qiu

**Affiliations:** ^1^ College of Mechanical and Electrical Engineering, Sichuan Agriculture University, Ya’an, China; ^2^ College of Resources, Sichuan Agriculture University, Chengdu, China; ^3^ School of Mechatronic Engineering and Automation, Foshan University, Foshan, China; ^4^ College of Biosystems Engineering and Food Science, Zhejiang University, Hangzhou, China; ^5^ Institute of Urban Agriculture, Chinese Academy of Agriculture Sciences, Chengdu, China; ^6^ School of Electrical and Information Engineering, Jiangsu University, Zhenjiang, China; ^7^ Sichuan Academy of Agricultural Machinery Sciences, Chengdu, China

**Keywords:** non-green-ripe citrus, multiripeness fruits, YOLOv7, EfficientNet, CBAM, agricultural robot

## Abstract

This study addresses the challenges of low detection precision and limited generalization across various ripeness levels and varieties for large non-green-ripe citrus fruits in complex scenarios. We present a high-precision and lightweight model, YOLOC-tiny, built upon YOLOv7, which utilizes EfficientNet-B0 as the feature extraction backbone network. To augment sensing capabilities and improve detection accuracy, we embed a spatial and channel composite attention mechanism, the convolutional block attention module (CBAM), into the head’s efficient aggregation network. Additionally, we introduce an adaptive and complete intersection over union regression loss function, designed by integrating the phenotypic features of large non-green-ripe citrus, to mitigate the impact of data noise and efficiently calculate detection loss. Finally, a layer-based adaptive magnitude pruning strategy is employed to further eliminate redundant connections and parameters in the model. Targeting three types of citrus widely planted in Sichuan Province—navel orange, Ehime Jelly orange, and Harumi tangerine—YOLOC-tiny achieves an impressive mean average precision (mAP) of 83.0%, surpassing most other state-of-the-art (SOTA) detectors in the same class. Compared with YOLOv7 and YOLOv8x, its mAP improved by 1.7% and 1.9%, respectively, with a parameter count of only 4.2M. In picking robot deployment applications, YOLOC-tiny attains an accuracy of 92.8% at a rate of 59 frames per second. This study provides a theoretical foundation and technical reference for upgrading and optimizing low-computing-power ground-based robots, such as those used for fruit picking and orchard inspection.

## 1 Introduction

Citrus is one of the most widely cultivated and highest-yielding fruit crops globally, generating significant economic value (The United States Department of Agriculture, 2024). However, the citrus industry faces immense pressure due to skilled labor shortages, rising production costs, market demand fluctuations, and extreme climate changes (Castro-Garcia et al., 2019; Apolo-Apolo et al., 2020a). Agricultural robots can mitigate these pressures by reducing reliance on skilled labor, lowering economic and environmental costs, and enhancing orchard management and productivity (Bargoti and Underwood, 2017; Fu et al., 2020a). Accurate fruit detection is essential for automated harvesting and early yield prediction (Zhuang et al., 2018; Apolo-Apolo et al., 2020b; Lu et al., 2023). Consequently, the detection of citrus fruits has become a research hotspot (Wang et al., 2022c; Ma et al., 2024). Particularly, there is an urgent need for high-performance detection models that can be deployed on resource-limited robots and other edge devices (Tang et al., 2020; Xu et al., 2023).

Multispectral cameras, optical digital cameras, 3D stereoscopic cameras, and RGB-D depth cameras are the primary devices used for fruit detection (Chen et al., 2020; Condotta et al., 2020). Multispectral cameras can capture spectral information across various bands from visible to near-infrared and are commonly mounted on unmanned aerial vehicles for large-scale crop health, yield estimation, and disease monitoring (Huang et al., 2020; Lan et al., 2020). However, their high cost and complex data processing requirements limit their application in ground-based agricultural robots. Optical digital cameras, 3D stereoscopic cameras, and RGB-D depth cameras typically produce RGB images with three visible light bands: red, green, and blue. Many studies have shown that RGB images are sufficient for fruit detection (Lu et al., 2018; Yu et al., 2019; Gené-Mola et al., 2020; Liu et al., 2023). These devices are cost-effective and require less computational power, making them more suitable for the practical needs of real-time monitoring and automated harvesting robots.

Over the past few decades, methods combining digital image processing with traditional machine learning (ML) techniques have been used for fruit detection, including citrus (Liu et al., 2018), kiwifruit (Fu et al., 2019), and apples (Lu et al., 2022). However, the pixel values in RGB images are highly sensitive to changes in lighting and background interference. Traditional ML algorithms, such as support vector machines and decision trees, rely on complex feature extraction and manual rules to handle these variations (Fu et al., 2018). Consequently, these algorithms exhibit performance fluctuations in complex environments and fail to meet the need for stable citrus fruit detection by robots in real-world scenarios.

In recent years, the advancement of deep learning (DL) technology has significantly impacted the field of agricultural detection due to its exceptional feature learning capability, robust generalization performance, and substantial computational power (Gené-Mola et al., 2020; Maheswari et al., 2021). DL methods for fruit detection are broadly categorized into two main approaches: region-based two-stage methods (Girshick et al., 2014; Shaoqing et al., 2016) and end-to-end single-stage methods (Redmon et al., 2016; Wei et al., 2016).

Two-stage detection methods first extract a large number of regions of interest (RoIs) that potentially contain target fruits. These RoIs are then passed through a convolutional neural network (CNN) for detection, with final detection results obtained after post-processing (Girshick et al., 2014; Shaoqing et al., 2016). Although this process is time-consuming, these methods typically achieve high detection precision due to the utilization of CNNs for fruit detection on RoIs (Redmon et al., 2016). For example, C.H. Yang et al. (Yang et al., 2020) developed a citrus fruit detection algorithm based on Mask R-CNN, achieving a detection precision of 88.15%. Longsheng Fu et al. (Fu et al., 2020b) proposed an apple detection algorithm based on Faster R-CNN, achieving a detection precision of 89.3%. However, the inherent characteristics of two-stage methods, including slower detection speed and high memory consumption, limit their suitability for applications such as harvesting robots, which require rapid detection and are constrained by computational resources.

In contrast, the YOLO series of single-stage detection methods, introduced in 2015, offers faster detection speeds and high detection accuracy (Redmon et al., 2016). YOLO models perform target detection in a single pass through a CNN, eliminating the need for separate stages and reducing redundant operations (Redmon et al., 2016; Wang et al., 2022a). While early YOLO models had lower detection accuracy compared to two-stage models like R-CNN, subsequent optimizations, and improvements by numerous researchers have led to the development of several highly effective fruit detection methods. For instance, Longsheng Fu et al. (Fu et al., 2021) proposed a kiwifruit detection algorithm, DY3TNet, by improving the YOLOv3-tiny model, achieving a detection precision of 90.05%. Shenglian Lu et al. (Lu et al., 2022) developed the CA-YOLOv4 detection algorithm for apples in orchard environments, achieving a detection precision of 92.6% for Envy apples during harvest. Additionally, Lijia Xu et al. (Xu et al., 2023) proposed the HPL-YOLOv4 citrus detection model for complex environments, achieving a detection precision of 93.45%.

Citrus is a general term for fruits belonging to the Citrus genus of the Rutaceae family, with major types including grapefruit, lemon, tangerine, and orange (Liu et al., 2012). Among these, navel oranges, Ehime Jelly oranges, and Harumi tangerines are widely cultivated in the southwestern regions of China, and their fruits turn orange-red upon ripening. In this study, we refer to them as non-green-ripe citrus. While existing models can detect single-variety or single-degree ripeness fruits, such as apples or certain citrus fruits (Lu et al., 2018, 2022), there remains an urgent need for a real-time and accurate detection model for multi-ripeness fruits of different non-green-ripe citrus varieties in complex orchards. To address this issue, we first collected and created a custom image dataset of non-green-ripe citrus, covering the detection needs of unripe, semi-ripe, and ripe fruits. We then proposed a lightweight, single-stage citrus detection model suitable for deployment on edge devices such as robots. The main contributions of this work are as follows:

(1) We designed a comprehensive image dataset, RC3025, which includes images of non-green-ripe citrus fruits of various varieties and ripeness levels in complex scenarios.
(2) We discovered and proposed that incorporating a small number of pure citrus fruit images into the training set enhances the model’s detection performance in real orchards.
(3) We developed a general, lightweight, and high-performance multi-ripeness citrus recognition model, YOLOC-tiny, based on YOLOv7.
(4) We validated the practical performance and advantages of YOLOC-tiny through robot deployment application experiments, demonstrating its effectiveness in detecting non-green-ripe citrus fruits in complex environments.

## 2 Materials and methods

### 2.1 Multiripeness non-green-ripe citrus fruit image dataset

#### 2.1.1 Raw image acquisition and labeling

**Figure 1:**
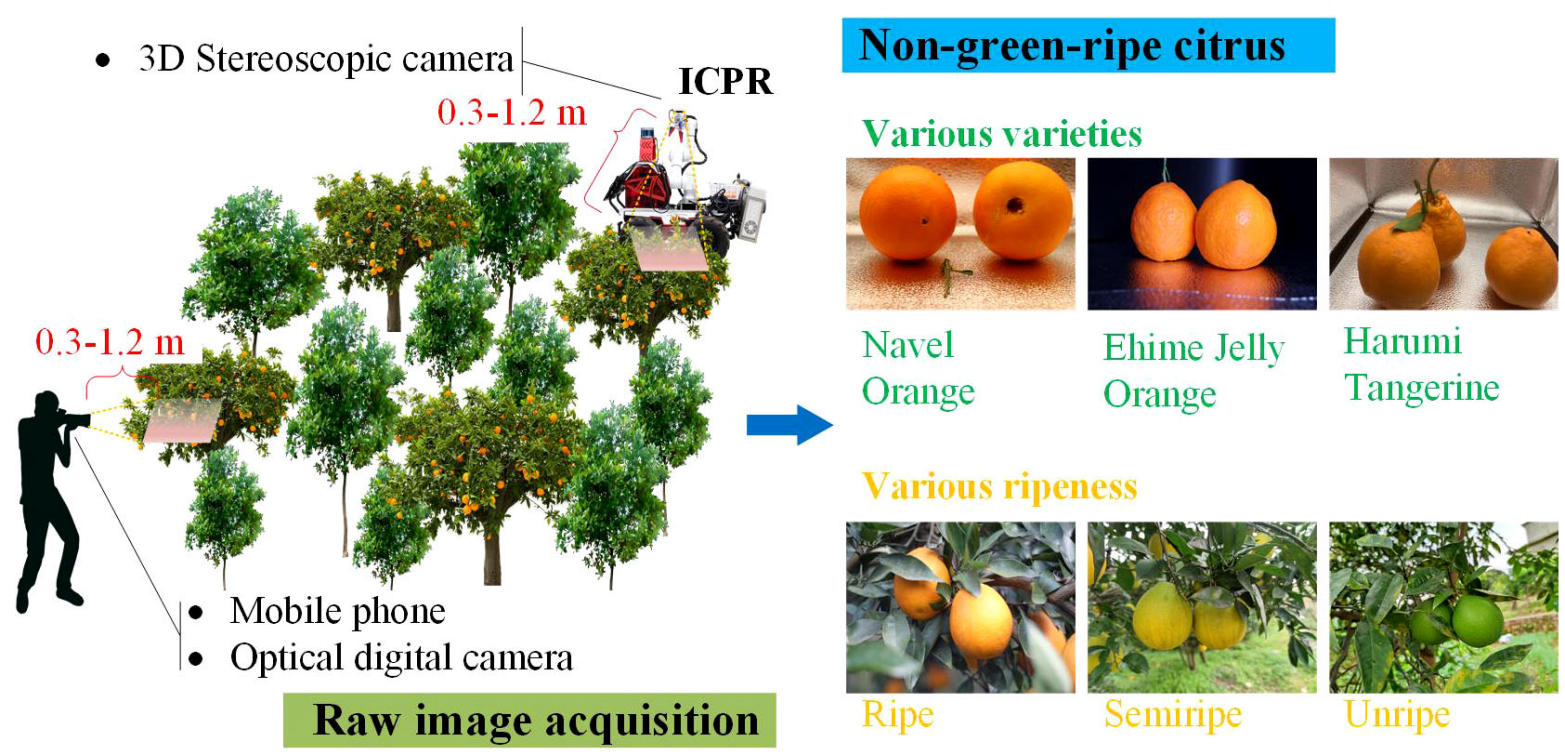
Schematic diagram of how to capture the images of non-green-ripe citrus fruits and some examples.

To properly train the developed DL model, a number of raw images are required as an initial dataset (Apolo-Apolo et al., 2020a). Research suggests that 2,500 annotated instances are adequate for training deep networks to recognize a certain type of fruit (Wang et al., 2022b). From 2020 to late 2023, we continuously collected raw images over four years using both manual and robotic photography, as shown in **Figure 1**. Various imaging devices, including a 3D stereoscopic camera (ZED), a Canon 80D camera, and four different mobile phones (VIVO Y97, Mi 10, Redmi K40, and iPhone Xs), were employed to capture images of citrus fruits at different ripeness levels and varieties in three non-green-ripe citrus orchards. These orchards are located in three different counties in the western part of Sichuan Province: Yucheng District, Ya’an City (29°58′N, 102°59′E); Jintang County, Chengdu City (30°43′N, 104°29′E); and Danling County, Meishan City (29°58′N, 103°32′E). To meet the operational needs of ground-based agricultural robots, the shooting distance ranged from 0.3 to 1.2 meters.

Experienced researchers screened the raw images and collected a total of 2,905 image data samples covering three citrus varieties (navel orange, Ehime Jelly orange, and Harumi tangerine) at different ripeness levels in unstructured orchards. Additionally, to investigate whether pure citrus images could enhance model detection performance, 120 images featuring pure citrus fruits were captured in the laboratory using both ZED and digital cameras. Specifically, the sections pertaining to citrus images in complex orchards and pure citrus images were separately labeled as RC2905 and RC120. The labeling process for the 3,025 images was completed using the open-source tool LabelImg (Tzutalin, 2015), and the citrus image dataset RC3025 (raw citrus dataset with 3,025 images) was created, comprising a total of 10,653 labeled instances. Details of the dataset are shown in **Supplementary Table 1**.

#### 2.1.2 Dataset partitioning

**Figure 2:**
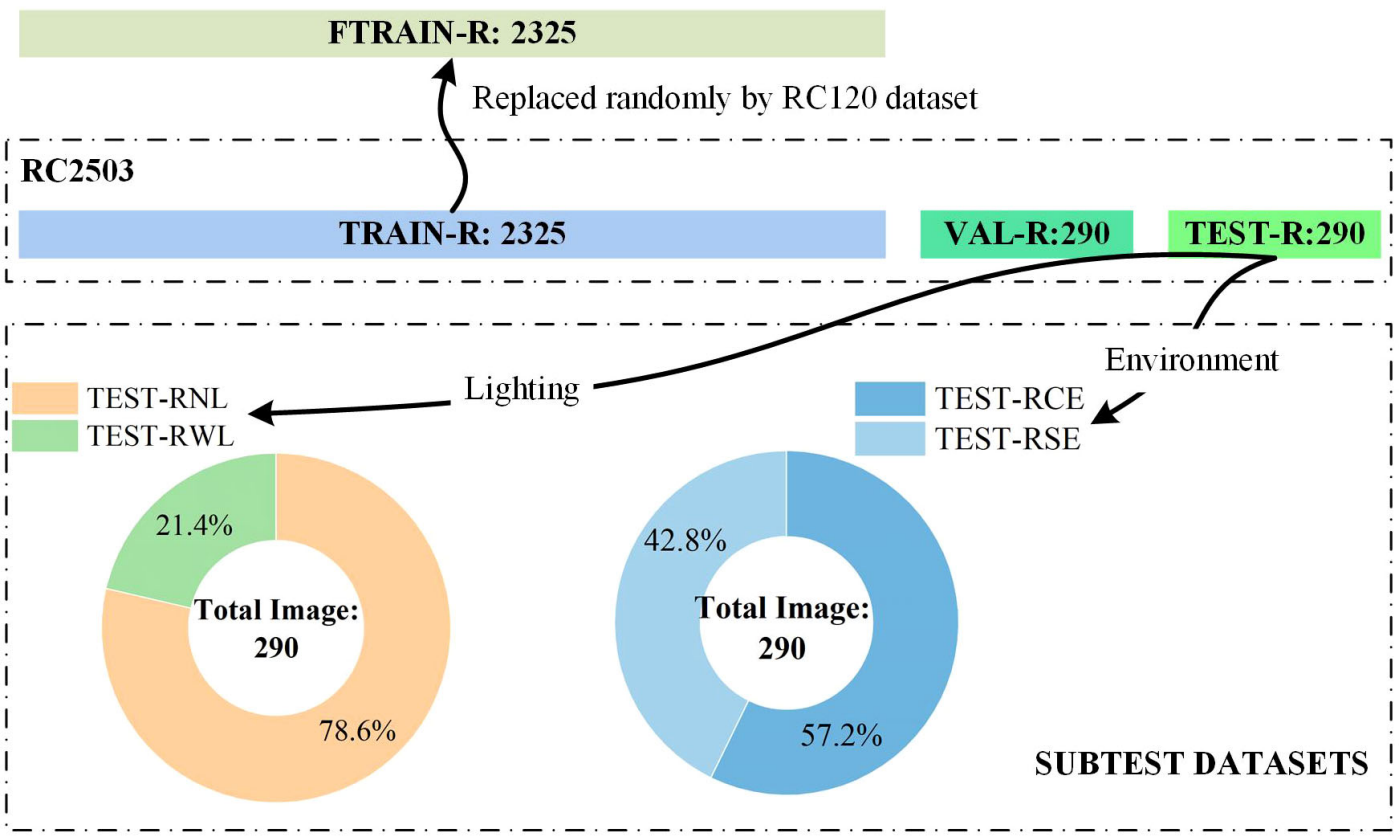
Diagram of dataset partitions.

Several studies have successfully used a 10% validation split (Lu et al., 2022; Liu et al., 2023; Xu et al., 2023), achieving significant detection results. To balance computational resources and maintain training efficiency, the RC2905 dataset was partitioned into the raw training set (TRAIN-R), the raw validation set (VAL-R), and the raw test set (TEST-R) in an 8:1:1 ratio, as illustrated in **Figure 2**. The RC120 dataset was employed to investigate the impact of pure citrus images on model performance by randomly substituting 120 images in TRAIN-R, defining the refined training set as FTRAIN-R after fine-tuning. TEST-R was further categorized based on background complexity and citrus occlusion, resulting in a complex background test set comprising 166 images (TEST-RCE) and a simple background test set with 124 images (TEST-RSE). Given the variations in light intensity, the test set was further stratified into a set containing 228 images with normal light intensity (TEST-RNL) and another set containing 62 images under low-light conditions (TEST-RWL).

#### 2.1.3 Image dataset augmentation

**Figure 3:**
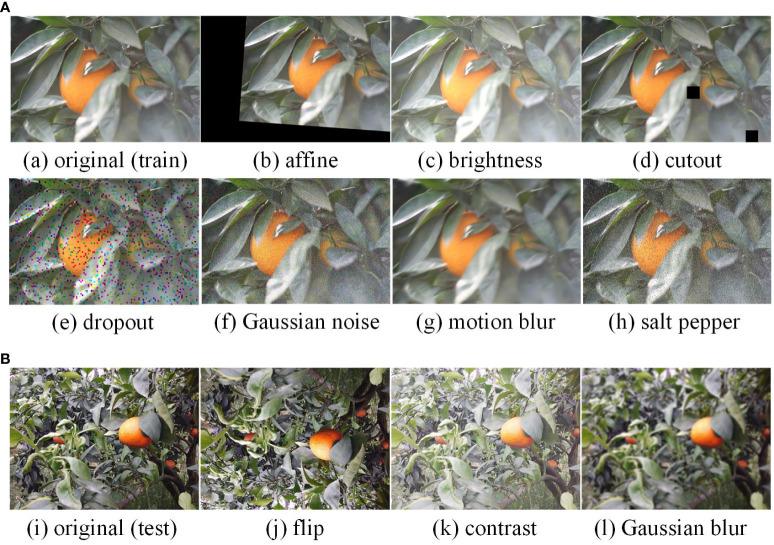
Image augmentations. **(A)** Augmentation methods for TRAIN-R, FTRAIN-R, and VAL-R: affine transformation, brightness adjustment, cutout, coarse dropout, Gaussian noise, motion blur, and salt and pepper noise. **(B)** Augmentation methods for TEST-R: up and down flip, contrast adjustment, and Gaussian blur.

Many studies demonstrated that enhancing raw images can improve the model’s generalization ability. In the present study, seven enhancement methods, including affine transformation, luminance adjustment, cut-out, coarse dropout, Gaussian noise, motion blur, and salt–pepper noise, were employed to augment the training and validation sets, as illustrated in **Figure 3**. These enhancement operations were executed on TRAIN-R, VAL-R, TEST-R and FTRAIN-R, resulting in the corresponding enhanced datasets TRAIN-A, VAL-A, TEST-A and FTRAIN-A, respectively. Three enhancement methods—up and down flip, contrast adjustment, and Gaussian blur—were simultaneously applied to the test set. Two datasets, TEST-ANL and TEST-AWL, were generated by enhancing the original test set of normal and weak light environments. Additionally, the TEST-ACE and TEST-ASE datasets were created by augmenting the original test sets of complex and simple environments, respectively. These enhanced datasets aim to simulate accurately the diverse lighting conditions, backgrounds, and fruit states in real-life orchard scenes, thereby bolstering the robustness and accuracy of the detection models in practical scenarios. An overview of the augmented dataset and the number of images is provided in **Supplementary Table 2**.

### 2.2 Design of the YOLOC-tiny model

Orchard operation robots face constraints due to limited computational resources, whereas traditional DL models pose challenges with their high computational complexity and demanding hardware requirements. To ensure that robots can reliably, accurately, and efficiently detect various types and ripeness levels of citrus fruits in complex non-green-ripe citrus orchards, we initially used our custom datasets TRAIN-A, VAL-A, and TEST-A to train and test most of the popular SOTA object detectors, including YOLOv7 and YOLOv8. Based on the practical needs of robotic operations and the experiment results, we chose YOLOv7 (Wang et al., 2022a) as the foundational network and conducted a series of optimizations and improvements.

Given the large size of the YOLOv7 backbone network, we selected a lightweight feature extraction network, EfficientNet-B0, to replace the original backbone. After comparing various advanced attention mechanisms, we introduced a composite efficient attention mechanism, CBAM, to enhance target perception. Subsequently, after numerous experiments, we carefully designed an extended efficient aggregation network module incorporating CBAM, called Efficient Layer Aggregation Networks in the Head with CBAM (ELAN-HC). Considering the phenotypic features of the targets, we proposed an adaptive and efficient complete intersection over the union regression loss function (ACIoU). This function allows for adjustments to the aspect ratio regression loss penalty factor, enhancing the perception ability of citrus fruits and consequently improving detection accuracy.

**Figure 4:**
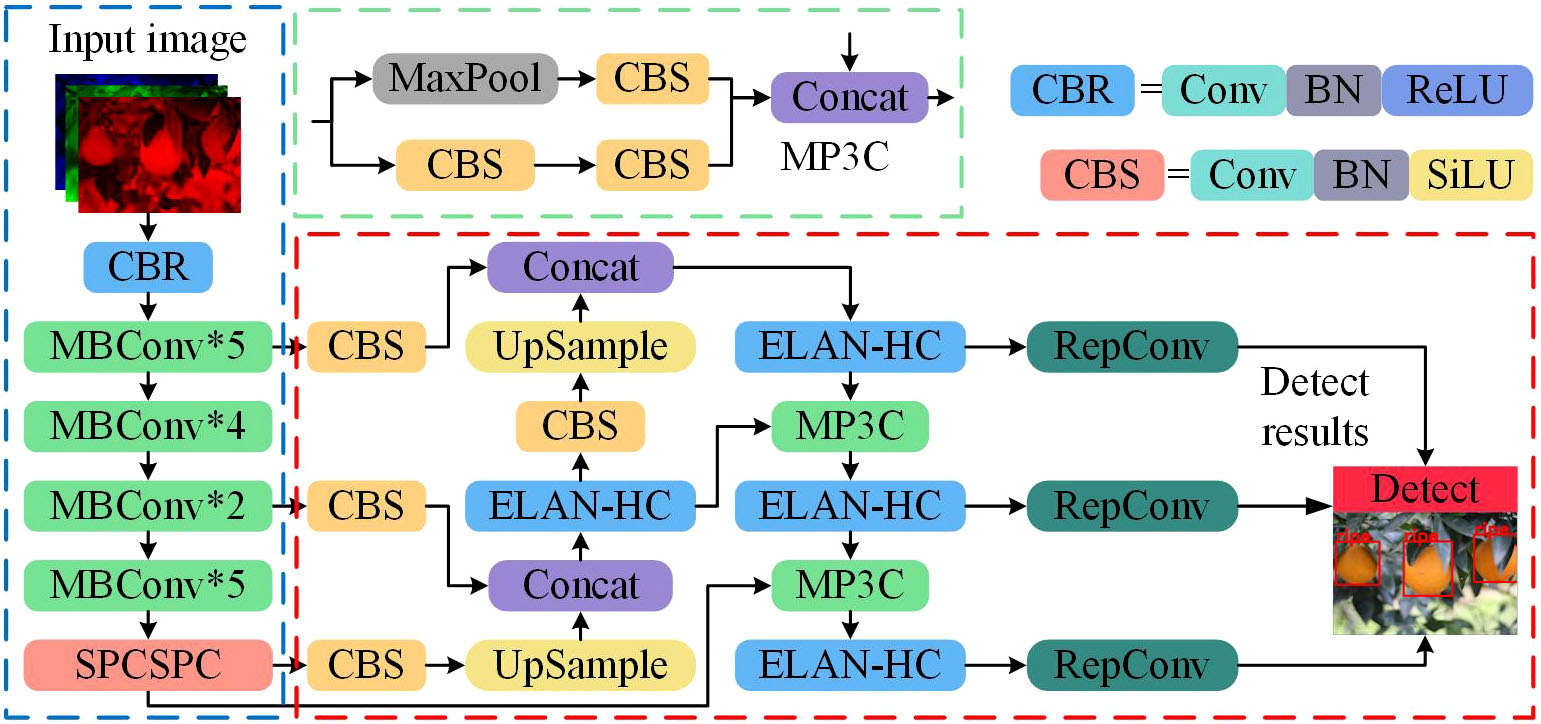
Structure of the YOLOC network.

We integrated these measures to develop a generalized base network, YOLOC, where C stands for citrus, to recognize non-green-ripe citrus varieties such as navel orange, Ehime jelly orange, and Harumi tangerine in complex environments, particularly in the hilly areas of southwest China. The structure of YOLOC is depicted in **Figure 4**, where RepConv denotes reparametrized convolution. Furthermore, we leveraged transfer learning to train YOLOC on FTRAIN-A and employed sparse training and Layer-based Adaptive Magnitude Pruning (LAMP), a quantized pruning technique, to derive a lightweight recognition model, YOLOC-tiny.

#### 2.2.1 ACIoU

The accuracy of target detection and localization is significantly influenced by the choice of the loss function (Yu et al., 2022). The loss function was computed based on the intersection over union (IoU), and the CIoU utilized by YOLOv7 comprehensively considered the variations in the overlap area, center distance, and aspect ratio between the predicted box and the ground truth box (Zheng et al., 2020), as illustrated in (Equations 1–3).


(1) $$L\text{o}ss_{CIoU}=1-IoU+\frac{\rho^{2}\left(\begin{array}{cc} b, & b^{gt} \end{array}\right)}{c^{2}}+\alpha v,$$

(2) $$\alpha =\frac{v}{1-IoU+v},$$

(3) $$v=\frac{4}{\pi^{2}}{\left(\arctan\frac{w^{gt}}{h^{gt}}-\arctan\frac{w}{h}\right)}^{2},$$

where $L\text{o}ss_{CIoU}$ denotes the loss value, IoU represents the IoU ratio between the ground truth box and the predicted box, $\rho^{2}\left(\begin{array}{cc} b, & b^{gt} \end{array}\right)$ signifies the Euclidean pixel distance between the ground truth box and the predicted, c represents the diagonal length of the smallest enclosing area that surrounds both the predicted and ground truth bounding boxes, α is the acquired trade-off coefficient, v denotes the consistency factor of the width and height of the predicted box and the ground truth box, $w^{gt}$ and $h^{gt}$ are the width and height of the ground truth box, respectively, and w and h are the width and height of the predicted box, respectively.

The CIoU loss function is commonly employed in target detection tasks; however, it exhibits the following drawbacks (Yu et al., 2022): (1) The use of an inverse tangent function in CIoU makes it highly sensitive to outliers, resulting in poor robustness. (2) The value domain $\left(\begin{array}{cc} 0, & \pi /2 \end{array}\right)$ of the inverse tangent function cannot directly fulfill the normalization requirements of the loss function. (3) Adaptability to adjust the corresponding features of the loss function based on the detection object is lacking. Hence, considering the phenotypic features of the large non-green-ripe citrus fruits, we proposed ACIoU. This function can dynamically adjust the length and width regression loss penalty factor based on the phenotypic parameters of citrus fruits, as depicted in (Equations 4–6).


(4) $$L\text{o}ss_{ACIoU}=1-IoU+\frac{\rho^{2}\left(\begin{array}{cc} b, & b^{gt} \end{array}\right)}{c^{2}}+\alpha \gamma ,$$

(5) $$s\left(\begin{array}{ccc} a, & b, & x \end{array}\right)=\frac{1}{1+e^{-a\left(x-b\right)}},$$

(6) $$\gamma ={\left(s\left(\begin{array}{ccc} a, & b, & \frac{w^{gt}}{h^{gt}} \end{array}\right)-s\left(\begin{array}{ccc} a, & b, & \frac{w}{h} \end{array}\right)\right)}^{2},$$

where $L\text{o}ss_{ACIoU}$ represents the value of the ACIoU function, a and denotes the adaptive Sigmoid deformation parameters that can be adjusted based on different aspect ratios of the detection targets, and γ signifies the adaptive consistency factor of the width and height of the predicted box and the ground truth box.

**Figure 5:**
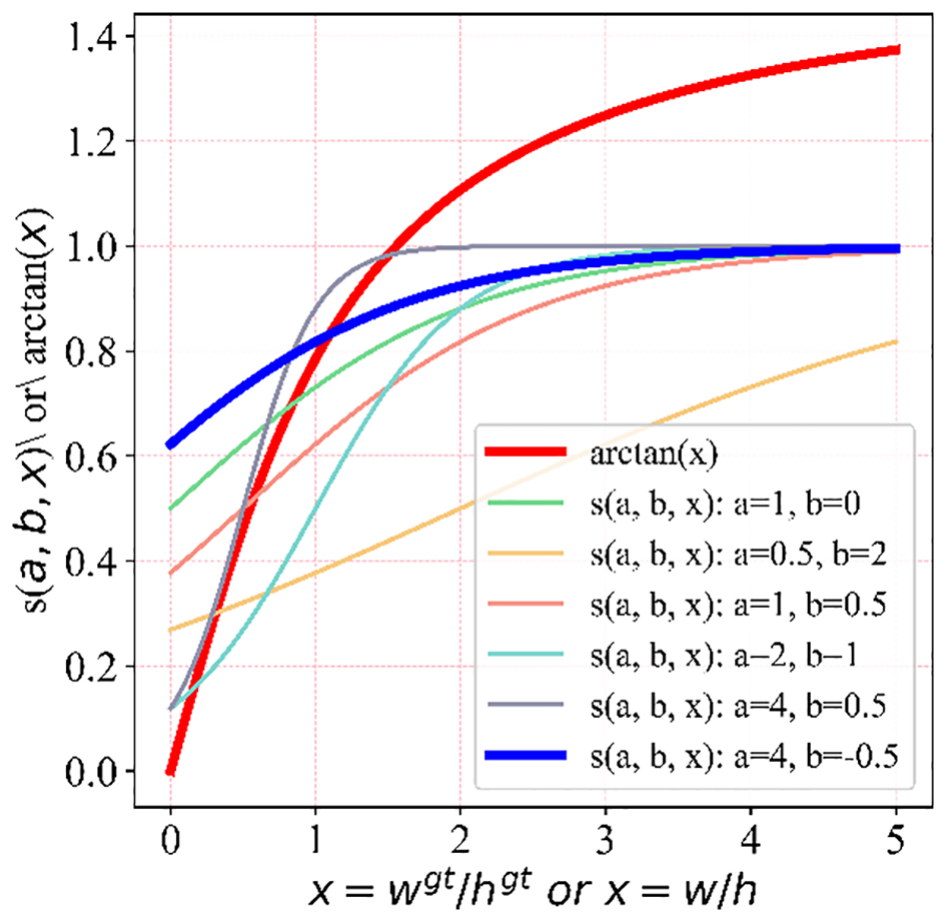
Loss penalty curves of the width and height differences with different deformation parameters.

**Figure 6:**
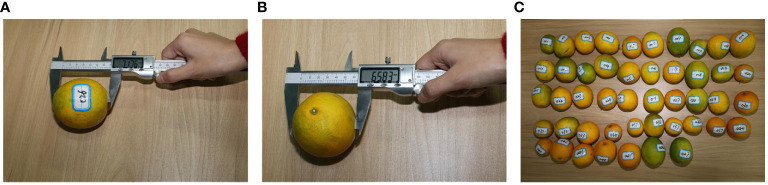
Measurement methods of longitudinal and transverse diameters and samples. **(A)** Measurement method of citrus fruit longitudinal diameter. **(B)** Measurement method of citrus fruit transverse diameter. **(C)** Samples of citrus fruits.

The variation curves of the width and height difference loss penalty terms corresponding to the real and predicted boxes for different deformation parameters, a and b , are presented in **Figure 5**. The disparity between the length and width of the ground truth box of citrus fruits is smaller than that in Microsoft Common Objects in Context (COCO). We randomly selected 47 citrus fruits with different maturity levels from the orchard of Ya’an Yucheng District, and their average transverse and longitudinal diameters were measured using vernier calipers. The transverse diameter represents the maximum equatorial diameter of the mandarin orange. The longitudinal diameter is the straight-line distance between the pith (at the stalk) and the center of the top of the fruit, as shown in **Figure 6**. The measured average longitudinal diameter of citrus was 68.04 mm, the average transverse diameter was 64.94 mm, and the average aspect ratio was 1.05. These measurements can serve as a reference for adaptively adjusting the loss function width and high consistency evaluation index.

#### 2.2.2 Efficient feature extraction backbone

This study utilized EfficientNet-B0 as the feature extraction backbone to optimize the model parameters for practical deployment in orchard robots for recognizing large non-green-ripe citrus. EfficientNet-B0, a lightweight and high-performance neural network, was designed using neural architecture search. The architecture primarily consists of mobile inverted bottleneck convolutions (MBConv), as illustrated in **Supplementary Figure 1**. MBConv integrates depthwise separable convolutions (DWConv) with Squeeze-and-Excitation (SE) blocks and inverse residual blocks. The SE module within MBConv dynamically recalibrates channel-wise feature responses by explicitly modeling interdependencies between the channels. With its DWConv and SE modules, MBConv offers a lightweight structure while maintaining good detection performance.

#### 2.2.3 ELAN-HC

**Figure 7:**
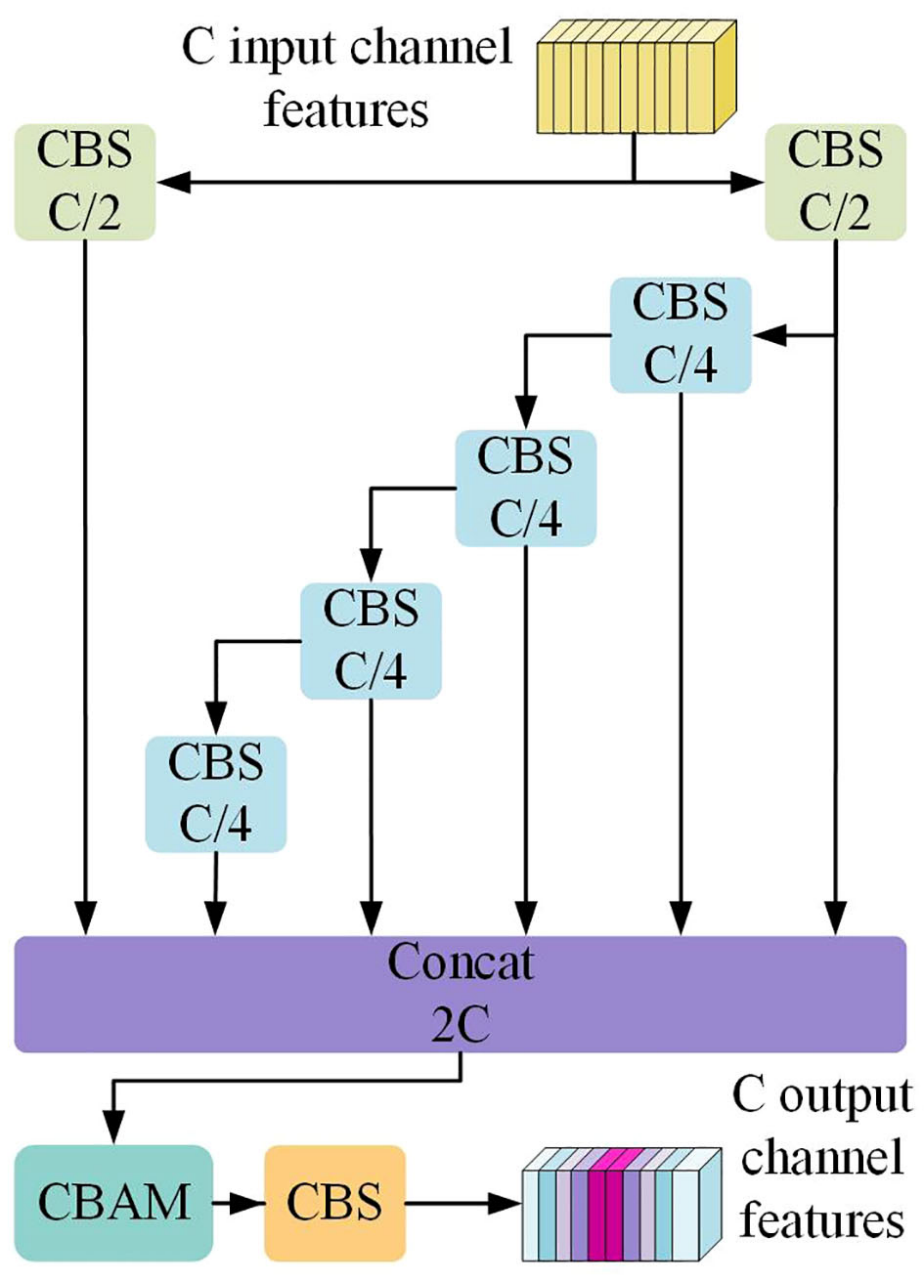
The structure of ELAN-HC.

The spatial attention mechanism amplifies the model’s capability to concentrate on specific regions within the image, facilitating the extraction of features crucial for target detection. The channel attention mechanism guides the model to prioritize significant features in the image, thereby contributing to an overall enhancement in target detection accuracy. CBAM integrates the channel attention mechanism and the spatial attention mechanism. YOLOv7 introduces efficient layer aggregation networks in the detection head (ELAN-H), leading to significant performance improvements. Empirically drawing on engineering experience, we incorporated CBAM into the ELAN-H network module, resulting in the formation of the ELAN-HC module, as depicted in **Figure 7**. This integration is aimed at optimizing further the model’s detection performance for non-green-ripe citrus in the unstructured orchards.

#### 2.2.4 Lightweight pruning strategy

We employed the LAMP pruning method on the trained YOLOC model to eliminate redundant parameters and connections, thereby enhancing the deployable performance and detection efficiency of YOLOC-tiny (**Supplementary Figure 2**). Subsequently, the pruned model underwent retraining in FTRAIN-A, resulting in the development of a lightweight detection model, YOLOC-tiny. The calculation for the LAMP score is expressed in Equation 7. The LAMP score was used to measure the importance of all weights in each layer of the YOLOC network to the citrus detection performance. During each round of pruning iterations, we removed the weights that contributed the least to the detection performance until the global sparsity constraint was satisfied. Thus, the model size was compressed, with little impact on its detection accuracy. LAMP retained at least one connection in each layer to ensure that at least one surviving connection was retained in each layer, thereby avoiding the loss of neurons and helping to maintain the ability to perceive non-green-ripe large citrus.


(7) $$score\left(u_{i}\right)=\frac{u_{i}^{2}}{\sum_{i\geq j}u_{j}^{2}},$$

where $u_{i}$ is the i th weight magnitude in the k th ( k=0,⋯359 ) layer of the YOLOC network after ascending order, and $score\left(u_{i}\right)$ is the LAMP score of $u_{i}$ .

## 3 Experiments and results

In this study, three computers, namely, PC1, PC2 and PC3, were employed for model training, testing, and deploying applications, respectively. PC3 is a lightweight industrial control mainframe computer integrated into the self-developed intelligent citrus picking robot (ICPR), as shown in **Figure 1**. The detailed hardware and software configurations of the three computers are provided in **Table 1**.

**Table 1:** Key hardware and software configurations of the experimental environment.

Hardware/ Software	PC1	PC2	PC3
CPU	Intel(R) Core(TM) i9-10920X CPU @ 3.50 GHz	Intel(R) Core(TM) i9-10920X CPU @ 3.50 GHz	Intel(R) Core(TM) i7-1165G7 CPU @ 2.80 GHz
GPUs	NVIDIA GeForce RTX 3090 (24576 MB) × 2	NVIDIA GeForce RTX 3090 (24576MB) × 2	NVIDIA GeForce MX450 (2048 MB)× 1
RAM	32 GB 3200 MHz × 4	32 GB 3200 MHz × 4	16 GB 3200 MHz × 1
Motherboard	ASUS WS X299 SAGE	ASUS WS X299 SAGE	HP 87E2
Operating system	Microsoft Windows 10 Pro (64-bit)	Microsoft Windows 10 Pro (64-bit)	Microsoft Windows 10 Pro (64-bit)
CUDA	11.7	11.8	11.8
cuDNN	8.5.0	8.7.0	8.7.0
PyTorch	2.0.0	2.0.1	2.0.1
OpenCV	4.7.0	4.8.0	4.8.0
Python	3.8.16	3.8.17	3.9.18
VS code	1.83.1	1.84.1	1.84.1

### 3.1 Model training and performance evaluation metrics

The model training was performed on PC1 and initialized with pre-trained weights from the COCO dataset. The stochastic gradient descent algorithm was used as the optimizer for model training. The training parameters included an initial learning rate of 0.01, momentum decay of 0.937, weight decay of 0.0005, a model input image size of 640 × 640, and a training epoch count of 300. A label smoothing strategy was implemented to address potential network overfitting resulting from incorrect data labeling by improving the model’s generalization ability. Additionally, online data augmentation using the mosaic method at each iteration was employed to enrich the citrus image data and further enhance the model’s generalization ability.

The model evaluation tests were conducted on PC2. The batch size for model test inputs was set to 1, the confidence threshold was 0.001, the IOU threshold was 0.6, and the model input image size was 640 × 640 by aligning with the practical conditions of the orchard robot. Given the constraints of the robot’s limited hardware resources, the models were comprehensively evaluated in this study based on three aspects, namely, basic detection performance, degree of lightweight, and detection speed, to assess the detection performance of different models. The evaluation of basic detection performance includes detection precision (P), recall (R), and mean average precision (mAP), which were calculated according to (Equations 8–10).


(8) $$P=\frac{TP}{TP+FP}\times 100\%,$$

(9) $$R=\frac{TP}{TP+FN}\times 100\%,$$

(10) $$mAP=\frac{1}{k}\sum_{i=1}^{k}\int_{0}^{1}P_{i}\left(R_{i}\right)d\left(R_{i}\right)\times 100\%,$$

where TP (true positive) represents the count of accurately detected citrus fruits, FP (false positive) signifies the count of erroneously identified objects or backgrounds as citrus fruits, FN (false negative) corresponds to the count of undetected or inaccurately identified citrus fruits, and k denotes the specific fruit type to be detected. In this study, k is 3, indicating the three categories of ripe, semi-ripe, and unripe citrus fruits.

The evaluation metrics for lightweight degree encompass the memory size occupied by the model (model size), the number of parameters (params), and the model detection speed measured by the number of FPS. Additionally, we introduced four normalized evaluation indicators, including the compound evaluator (CEval), which provides a holistic assessment of the model considering basic performance, the degree of lightweight, and detection speed. The CEval, model size score, model parameter score, and frame rate score are calculated as depicted in (Equations 11–14).


(11) $$CEval=\alpha_{1}P+\alpha_{2}mAP+\alpha_{3}SizeScore+\alpha_{4}\mathrm{Params}\text{Score}+\alpha_{5}FPSScore,$$

(12) $$SizeScore=\frac{1}{1+\exp\left(0.1\times \left(ModelSize-t_{1}\right)\right)},$$

(13) $$ParamsScore=\frac{1}{1+\exp\left(0.1\times \left(Params-t_{2}\right)\right)},$$

(14) $$FPSScore=\frac{1}{1+\exp\left(-\left(FPS-t_{3}\right)\right)},$$

where $\alpha_{1}$ , $\alpha_{2}$ , $\alpha_{3}$ , $\alpha_{4}$ , and $\alpha_{5}$ are the weight coefficients, and their sum is 1.0. They differentiate the importance of various indicators for intelligent operation robots in orchards. $t_{1}$ , $t_{2}$ and $t_{2}$ control the thresholds for each evaluation indicator.

**Figure 8:**
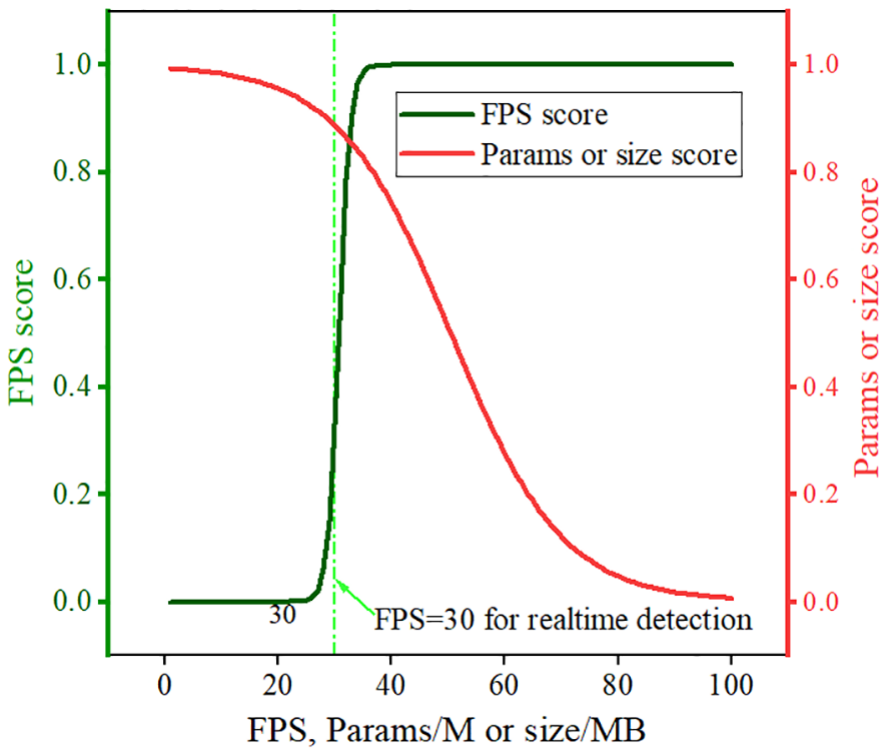
Evaluation metric score curves of model performance.

The curves illustrating the model size score, model parameter score, and frame rate score are presented in **Figure 8**. Slight variations in the FPS of each model were observed across experiments; thus, the FPS rates of all models were averaged over five tests after completing the graphics card warm-up. Aligned with the real-time target detection task and the goal of maintaining lightweight models, the threshold values (*t*_1_, *t*_2_, and *t*_3_) in this study were set at 50, 50, and 30, respectively. A high frame rate score indicates proximity to 1. However, the frame rate beyond the robot’s real-time monitoring need of 30 FPS becomes barely crucial. Conversely, parameter and model size scores approach 1 as they decrease and approach 0 as they increase.

### 3.2 Comparative experiments of different attention mechanisms

YOLOv7 was employed as a baseline to elucidate the impact of attention mechanisms on the detection performance of the YOLOC model. Various attention mechanisms were incorporated into the ELAN-H module, and comparative experiments were conducted to assess the detection performance for non-green-ripe large citrus. The results are presented in **Table 2**.

**Table 2:** Experimental results of incorporating various attention mechanisms.

Model	P/%	mAP/%	Size/MB	Params/M	FPS
YOLOv7	82.6	81.3	71.3	36.5	91
YOLOv7+CA	84.3	81.9	72.5	37.1	86
YOLOv7+ECA	83.5	82.2	71.3	36.5	90
YOLOv7+SE	80.8	82.1	72.8	37.3	91
YOLOv7+SimAM	83.6	82.0	71.3	36.5	89
YOLOv7+CBAM	83.8	82.5	72.9	37.3	86

All attention mechanisms were implemented in the same position within ELAN-H. CA refers to coordinate attention, ECA refers to efficient channel attention, and SimAM refers to a simple and effective attention module.

**Figure 9:**
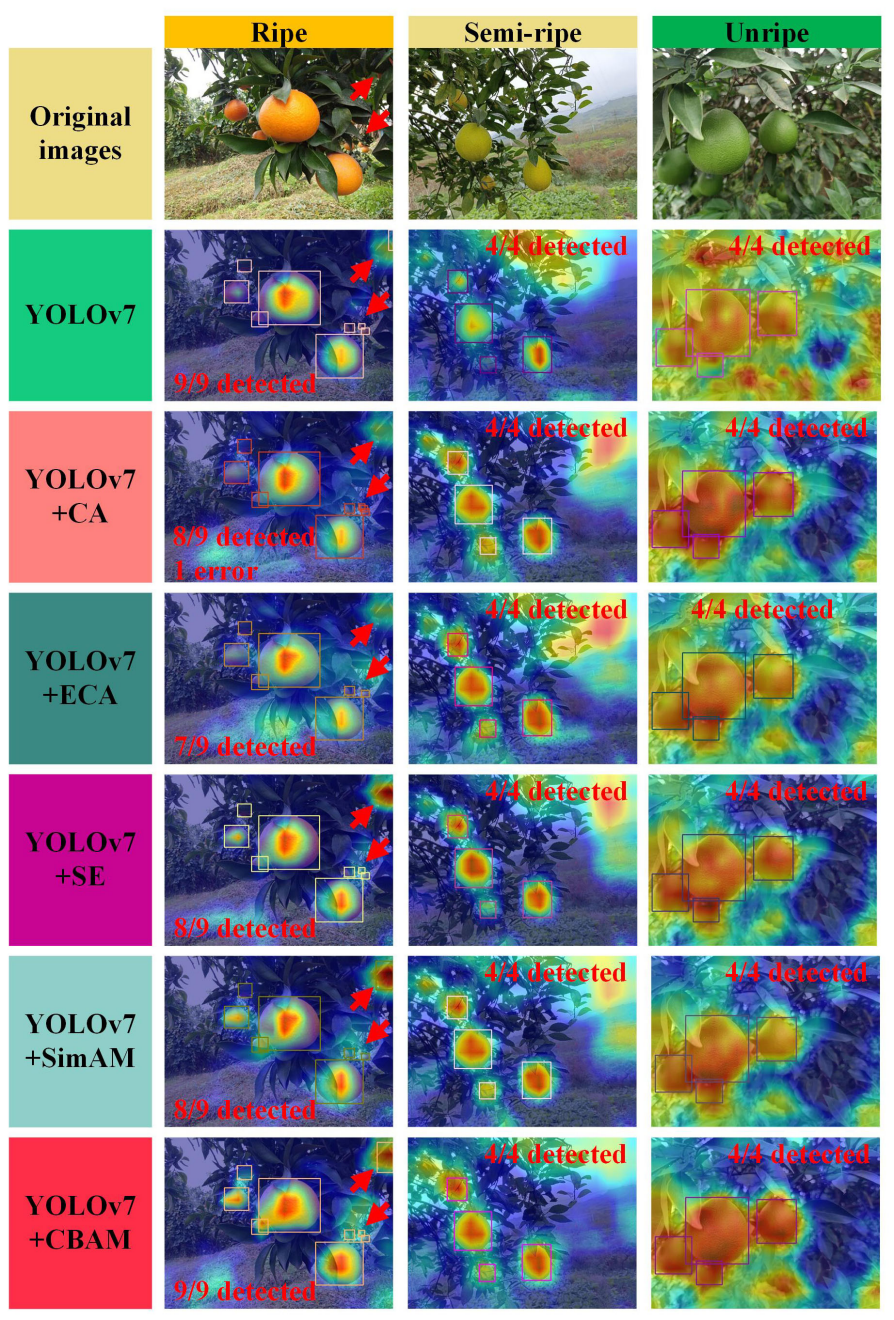
Thermograms and detection results of models integrated with various attention mechanisms. The red arrows indicate the locations where the detection results of different detectors are significantly different.

**Table 2** reveals that the introduction of different attention mechanisms impacts the model’s detection performance to varying degrees. Regarding performance metrics, YOLOv7+CBAM exhibits a detection accuracy of 83.8%, marking a 1.2 percentage point improvement over YOLOv7. Thus, it only ranks second to the YOLOv7+CA model. Compared with the average accuracy of YOLOv7, that of YOLOv7+CBAM reaches 82.5%, indicating a 1.2 percentage point increase, whereas that of YOLOv7+CA is only 81.9%. This finding suggests that YOLOv7+CBAM excels in capturing citrus image features. Although the model size and number of parameters of YOLOv7+CBAM experience a slight increase compared with those of YOLOv7, its detection accuracy is enhanced. The frame rate of YOLOv7+CBAM is 86 FPS, satisfying the real-time target detection requirements. We employed the GradCAM algorithm to generate detection heat maps for multiripeness citrus images and gain deep insights into the suitability of the CBAM attention mechanism in citrus fruit detection. The corresponding detection results are presented in **Figure 9**. All heat maps were generated at the same layer above the detection head of the detect network layer of the model.

**Figure 9** shows that different attention mechanisms allocate varying degrees of focus to citrus fruits, leading to differences in the detection performance of fruits at various ripeness levels. YOLOv7+CBAM exhibits the highest attention to citrus fruits with diverse ripeness, surpassing the attention given by YOLOv7, which allocates minimal attention to citrus fruits. For green unripe citrus, YOLOv7 distributes attention across the surroundings evenly. Despite the improvement in the model’s attention to citrus fruits with the introduction of other attention mechanisms, it still falls short of the performance achieved by the YOLOv7+CBAM model.

In terms of detection results, YOLOv7+CBAM and YOLOv7 exhibit no misdetections or omissions. By contrast, YOLOv7+CA has one omission and one misdetection in ripe citrus detection, YOLOv7+ECA has two omissions, and YOLOv7+SE and YOLOv7+SimAM have one omission in ripe citrus detection. In summary, the CBAM attention mechanism demonstrates superior performance in detecting multiripeness citrus fruits, particularly for unripe green citrus. Thus, it maintains high-precision results with no false or missed detections. Therefore, the CBAM attention mechanism proves to be well-suited for citrus fruit detection.

### 3.3 Ablation experiments

We conducted ablation experiments to assess comprehensively the impact of various enhancement measures on the model’s detection performance by incrementally introducing these measures with YOLOv7 as the baseline. The experimental results are presented in **Table 3**.

**Table 3:** Results of ablation experiments.

Model	P (%)	mAP (%)	Size (MB)	Params (M)	FPS
YOLOv7	82.6	81.3	71.3	36.5	91
YOLOv7+FTRAIN-A	85.0	82.2	71.3	36.5	90
YOLOv7+FTRAIN-A+EfficientNet-B0	85.5	82.1	23.2	11.7	88
YOLOv7+FTRAIN-A+EfficientNet-B0+CBAM	85.7	83.1	23.8	12.0	81
YOLOv7+FTRAIN-A+EfficientNet-B0+CBAM+ACIoU+ACIoU (YOLOC)	85.2	83.5	23.8	12.0	87
YOLOv7+FTRAIN-A+EfficientNet-B0+CBAM+ACIoU+LAMP+ACIoU+LAMP (YOLOC-tiny)	85.3	83.0	8.4	4.2	80

**Table 3** reveals that all proposed enhancements in this study lead to varying degrees of improvement in the model’s detection accuracy or lightweight characteristics. Compared with the training set without the use of the fine-tuned training set, the YOLOv7 model with the fine-tuned training set FTRAIN-A exhibits a 2.4% improvement in P and a 0.9% improvement in mAP. Furthermore, incorporating the lightweight backbone network EfficientNet-B0 further enhances the model’s detection accuracy and increases its lightweight profile. Compared with YOLOv7, the model with the EfficientNet-B0 backbone shows a 2.9% increase in P and a 0.8% increase in mAP value whilst maintaining only 32.5% and 32.1% of the model size and number of parameters of YOLOv7, respectively.

The introduction of the ELAN-HC module with the CBAM attention mechanism leads to a slight increase in model size and a decrease in frame rate. However, the P and mAP of the model show significant improvements, reaching 85.7% and 83.1%, respectively, representing a 3.1% and 1.8% increase compared with those of YOLOv7. Given these improvements, the YOLOC model with ACIoU experiences a marginal decrease in detection accuracy by 0.5% but improves in mAP and frame rate by 0.4% and 6 FPS, respectively.

The YOLOC-tiny model, derived through pruning and retraining on top of YOLOC, excels not only in accuracy but also in achieving an extremely compact model size. In particular, the P and mAP of the model are 85.3% and 83.0%, respectively, representing 2.7% and 1.7% increases compared with those of YOLOv7. The model size of YOLOC-tiny is 8.4 MB, with only 11.8% and 11.5% of the model size and number of parameters of YOLOv7, respectively.

### 3.4 Comparison experiments of different detectors

YOLOC-tiny was compared with the leading SOTA target detection models. The experimental results are presented in **Table 4**.

**Table 4:** Experimental results of different SOTA models.

Model	P/%	mAP/%	Size/MB	Params/M	FPS	Total Score
YOLOv5n	82.3	80.0	3.6	1.8	97	4.60
YOLOv5s	85.6	79.7	13.6	7.0	91	4.61
YOLOv5m	83.4	80.0	40.1	20.9	92	4.31
YOLOv5l	83.4	78.9	88.4	46.1	84	3.24
YOLOv5x	86.0	79.8	165.0	86.2	70	2.67
YOLOv6n	85.4	81.1	10.0	4.6	24	3.99
YOLOv6s	85.0	81.2	38.7	18.5	20	3.65
YOLOv6m	85.1	82.8	72.5	34.8	23	2.93
YOLOv6l	83.1	83.0	114.0	59.5	22	2.37
YOLOv7-tiny	80.6	82.1	11.6	6.0	101	4.59
YOLOv7	82.6	81.3	71.3	36.5	91	3.54
YOLOv7x	84.6	81.8	135.0	70.8	86	2.77
YOLOv8n	81.8	81.2	5.9	3.0	114	4.61
YOLOv8s	85.4	81.5	21.4	11.1	117	4.59
YOLOv8m	84.3	81.8	49.5	25.8	103	4.09
YOLOv8l	85.0	81.8	83.5	43.6	79	3.35
YOLOv8x	86.0	81.1	130.0	68.1	62	2.77
YOLOC	85.2	83.5	23.8	12.0	87	4.59
YOLOC-tiny	85.3	83.0	8.4	4.2	80	4.65

**Table 4** reveals that YOLOC-tiny achieves an accuracy of 85.3% in detecting multiripeness citrus fruits. This finding indicates that YOLOC-tiny outperforms most SOTA models and even surpasses YOLOC. Additionally, YOLOC-tiny attains an 83.0% mAP, ranking only second to YOLOC. YOLOC-tiny occupies a mere 8.4 MB of storage space, making it significantly more lightweight than YOLOv7x and YOLOv8x. The model’s parameter count is only 4.2 M, rendering it suitable for deployment in edge devices and resource-limited environments. Furthermore, YOLOC-tiny achieves a frame rate of 80 FPS, surpassing YOLOv8l and YOLOv8x. Thus, it is well-suited for real-time performance-critical scenarios.

**Figure 10:**
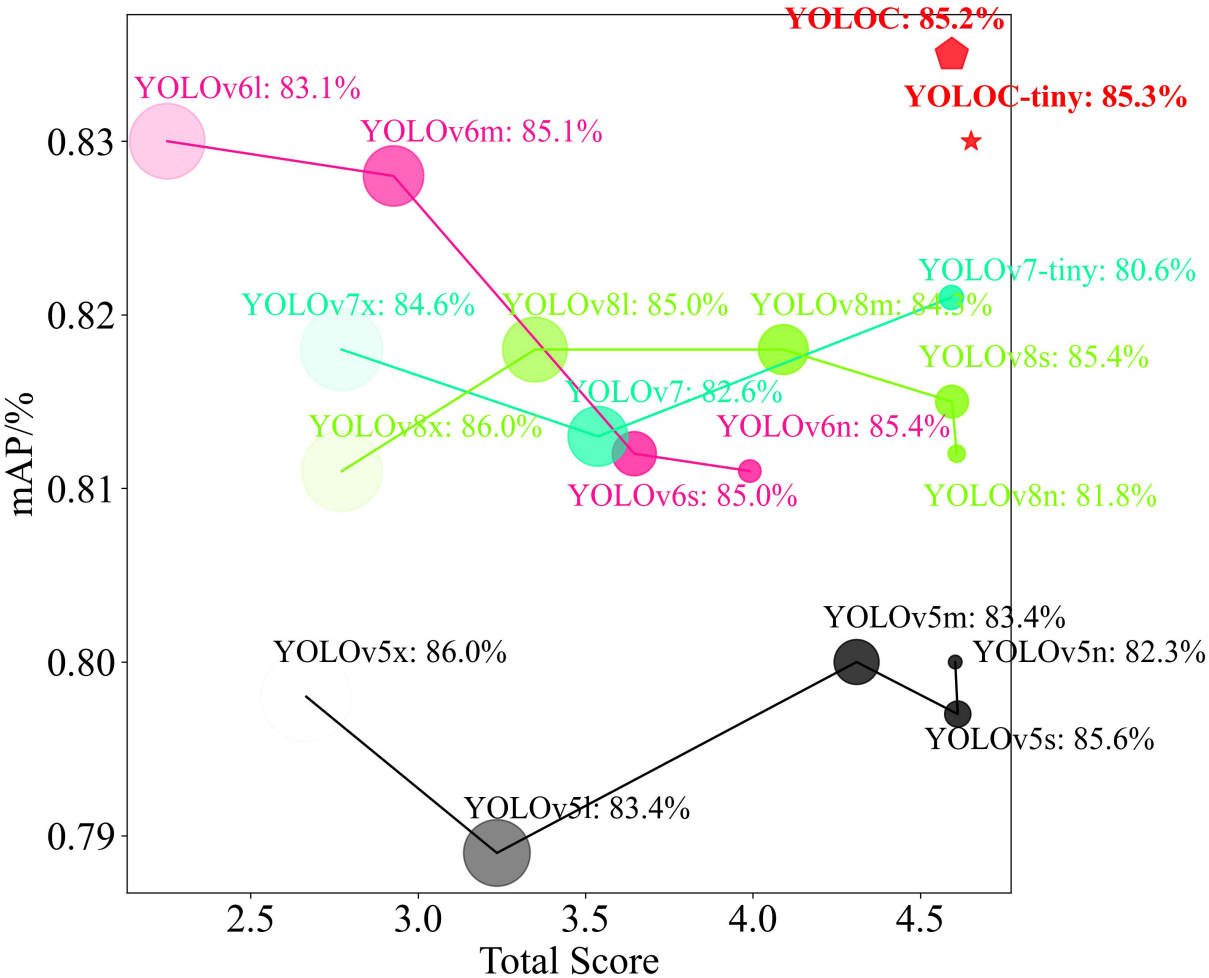
Detection performance charts for various SOTA models. The size of each geometric shape corresponds to the model size, with large shapes indicating large model sizes. The darkness of the geometric shape color represents the model parameter score, with dark colors indicating high parameter counts. The detection precision on the test set is provided for each model following its respective name.

We utilized the previously mentioned model lightweight, frame rate, and comprehensive performance indexes to analyze the detection performance of YOLOC series models comprehensively and thoroughly in complex environments for multiripeness and multispecies citrus fruits. The comprehensive performance diagrams of the aforementioned models were plotted, as shown in **Figure 10**. **Figure 10** shows that YOLOC and YOLOC-tiny exhibit excellent detection performance for citrus fruits. YOLOC and YOLOC-tiny demonstrate commendable average detection accuracies, with YOLOC-tiny being more compact than other models. This compactness contributes to reduced storage requirements on edge devices. YOLOC-tiny outperforms all other SOTA models, including YOLOC, in terms of the total score. Therefore, YOLOC-tiny has significant advantages in various aspects, including detection accuracy, lightweight design, frame rate, and overall performance. It exhibits the strongest overall performance, making it highly suitable for target detection in citrus orchard scenarios.

### 3.5 Comparison experiments in different environments

We extensively validated the YOLOC and YOLOC-tiny models across multiple validation subsets, encompassing various scenarios, to address varying lighting conditions and environmental complexities. These subsets comprise the test subsets TEST-ANL and TEST-AWL for diverse lighting conditions, along with the test subsets TEST-ACE and TEST-ASE representing varying environmental complexities. **Table 5** presents the average detection accuracies of different SOTA detectors on the respective test sets.

**Table 5:** Detection accuracy of SOTA detectors in different validation subsets.

Model	mAP/% (TEST-ANL)	mAP/% (TEST-AWL)	mAP/% (TEST-ACE)	mAP/% (TEST-ASE)
YOLOv5n	81.1	90.6	79.3	86.0
YOLOv5s	80.9	91.7	79.2	83.9
YOLOv5m	81.0	89.4	79.4	83.5
YOLOv5l	80.1	90.1	77.4	83.4
YOLOv5x	80.4	90.9	79.1	83.5
YOLOv6n	82.1	87.3	82.1	82.1
YOLOv6s	82.2	90.8	82.2	82.2
YOLOv6m	83.8	85.8	83.8	83.8
YOLOv6l	83.9	89.0	83.9	83.9
YOLOv7-tiny	83.0	88.5	81.9	85.1
YOLOv7	82.0	92.4	81.1	84.5
YOLOv7x	82.8	90.7	81.6	84.2
YOLOv8n	81.9	91.6	80.4	84.8
YOLOv8s	81.7	91.0	80.9	83.9
YOLOv8m	82.6	91.0	81.4	85.0
YOLOv8l	82.8	90.8	81.3	85.3
YOLOv8x	81.5	91.5	80.2	84.6
YOLOC	84.7	91.3	83.9	84.7
YOLOC-tiny	84.0	90.7	82.6	85.4

**Table 5** reveals that YOLOC-tiny exhibits notable mAP performance across all test sets, with impressive results on TEST-ANL and TEST-ACE. It achieves a substantial advantage on TEST-ANL, boasting a mAP of 84.0%, slightly below YOLOC’s 84.7%. This finding suggests that YOLOC-tiny excels in detection under regular lighting conditions. On TEST-AWL, the mAP of YOLOC-tiny is slightly lower than that of some algorithms. However, it still maintains a high level of performance. YOLOC-tiny achieves mAP scores of 85.4% and 82.6% on TEST-ASE and TEST-ACE, respectively, indicating its robustness in complex environments. These experimental results underscore the strong adaptability and practicality of YOLOC-tiny across various application scenarios.

### 3.6 Performance assessment in practical applications

**Figure 11:**
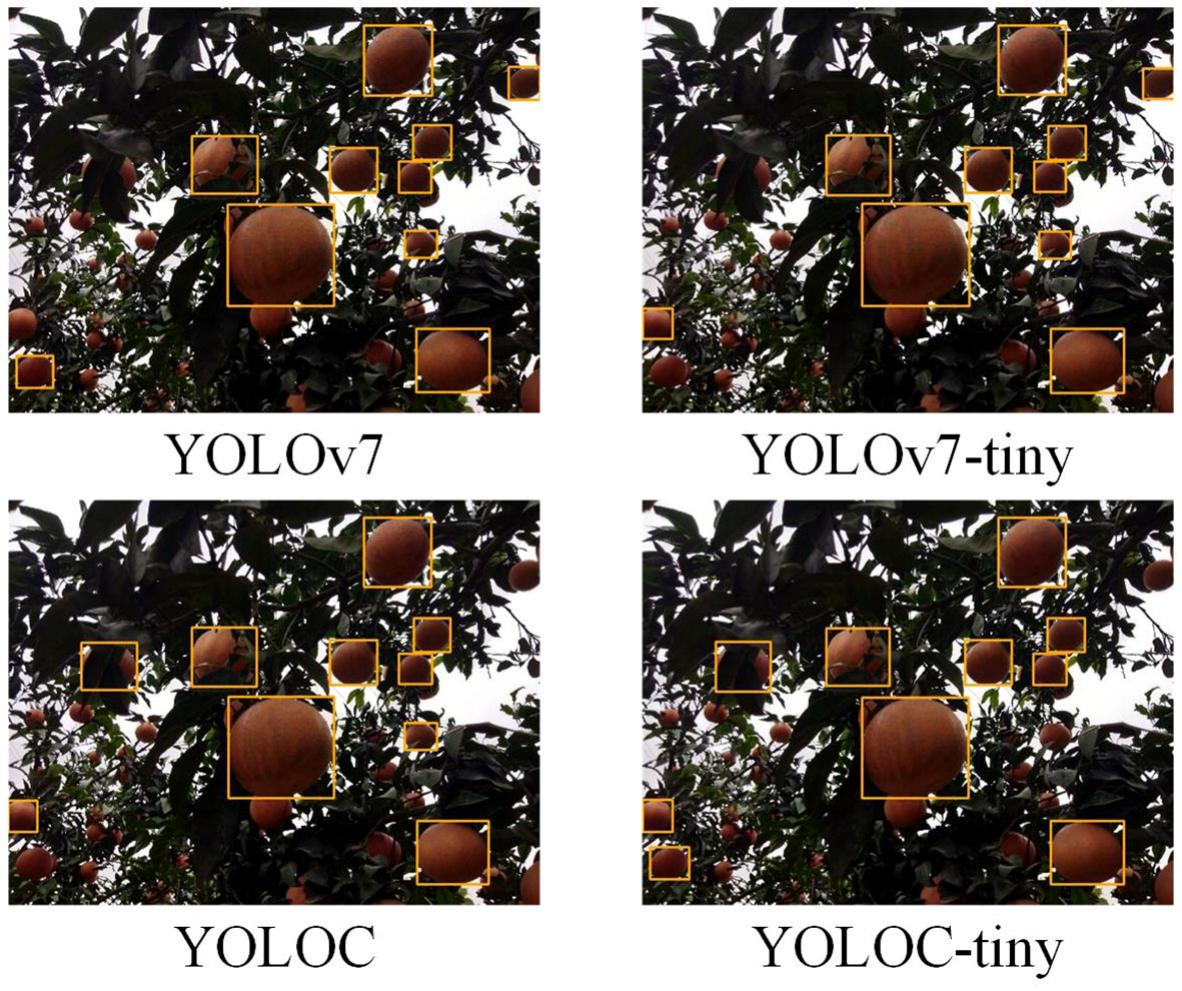
Detection results of various models in dark, complex environments.

Comparative experiments for real-world applications involving the YOLOC, YOLOC-tiny, YOLOv7, and YOLOv7-tiny models were conducted on ICPR, with deployment tests performed on PC3. The necessary software for model deployment includes onnx 1.14.0, onnxruntime-gpu 1.51.1, onnx-simplifier 0.4.33, and tensorrt 8.5.3.1. We initially exported the PyTorch models as general-purpose network models in the ONNX format. Then, we exported the ONNX model as a TensorRT model for ICPR deployment. Specific parameters, such as a confidence threshold of 0.4, an IOU threshold of 0.5, a model input image size of 640 × 640, and 32-bit floating-point precision, were set. Detection and labeling of images in the TEST-A dataset were performed on PC3 (**Figure 11**). Key metrics, including inference time, frame rate, detection accuracy, and the number of correctly detected citrus, were recorded. The accuracy rate was derived by sampling 29 images from the 290-image TEST-R test set for detection and manually verifying them multiple times. The detailed results are presented in **Table 6**.

**Table 6:** Results of robot application experiments.

Model	YOLOv7	YOLOv7-tiny	YOLOC	YOLOC-tiny
Inference time/ms	78.1	12.7	27.2	17.1
FPS	13	79	37	59
Accuracy/%	93.8	91.5	92.9	92.8
Number of citrus	3723	3801	3758	3852

The accuracy values in the table were calculated by comparing the model’s output results with the manual detection results.

**Figure 12:**
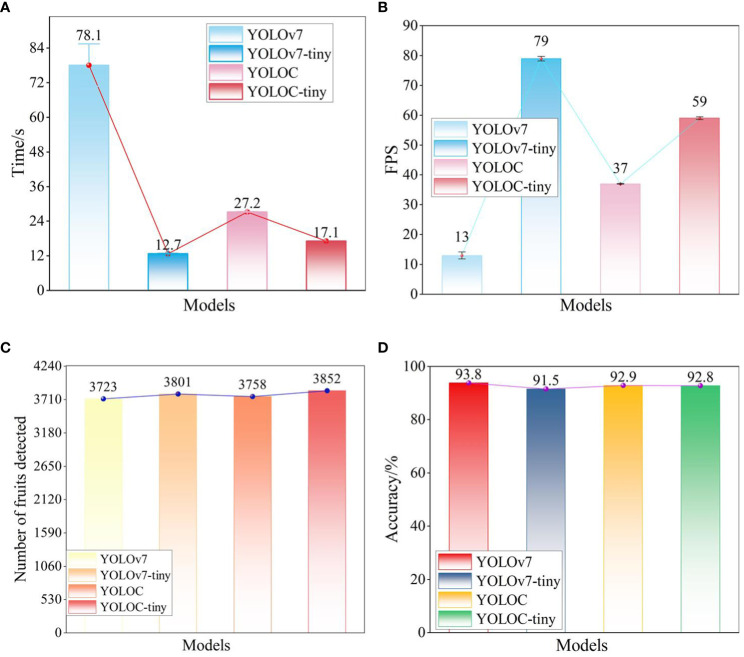
Detection results of different models deployed on ICPR. **(A)** Inference time of each model. **(B)** FPS of each model. **(C)** Number of citrus fruits detected. **(D)** Accuracy of each model.

**Table 6** reveals that the inference times for YOLOC and YOLOC-tiny are 27.2 and 17.1 ms, respectively. Although slightly higher than the 12.7 ms of YOLOv7-tiny, the values mentioned are significantly lower than the 78.1 ms of YOLOv7 (**Figure 12A**). YOLOC and YOLOC-tiny achieve frame rates exceeding the 30 FPS threshold required for the real-time detection needs of the robot, with YOLOC-tiny reaching an impressive 59 FPS. Although YOLOv7 demonstrates high detection accuracy, its FPS falls far below real-time requirements (**Figure 12B**). YOLOC-tiny detects 3852 citruses, outperforming the other three models (**Figure 12C**). This finding indicates its ability to capture targets comprehensively. Moreover, YOLOC-tiny exhibits superior real-time performance in citrus fruit detection. The detection accuracies of YOLOC and YOLOC-tiny are 92.9% and 92.8%, respectively, slightly lower than the detection accuracy of YOLOv7 (93.8%) but higher than that of YOLOv7-tiny (91.5%). This finding suggests that both models boast high detection accuracy and offer fast inference speeds and a good balance (**Figure 12D**). These experimental results further confirm the exceptional performance of the YOLOC series in real robotics applications.

## 4 Discussion

Detecting and localizing fruits are crucial for the agronomic management of fruit crops, including yield prediction and automated harvesting (Fu et al., 2020a; Lu et al., 2023). Fruit harvesting operations typically account for 25% of the total production cost and 50% of the total labor force (Castro-Garcia et al., 2019). Developing lightweight, high-precision detection models suitable for deployment on robots with limited computational power can ensure operational efficiency in complex orchard environments (Liu et al., 2023; Xu et al., 2023). This also can provide stable visual information for early yield prediction and fruit thinning operations.

Although excellent algorithms for detecting ripe fruits such as citrus fruits (Xu et al., 2023), apples (Wang and He, 2021), and kiwifruit (Fu et al., 2021), and for detecting apples at different growth stages (Ma et al., 2024), have been proposed, rapid detection of multi-variety and multi-ripeness citrus fruits in complex orchards remains challenging. Additionally, balancing detection performance, speed, and model parameters on edge devices with limited computational power has yet to be achieved satisfactorily.

Based on engineering experience and experimental results, we compared and analyzed various SOTA object detectors. We selected YOLOv7 as the base network and implemented a series of optimizations and improvements, including using a lightweight backbone network and embedding the attention mechanism CBAM. We also designed metrics to comprehensively evaluate the model’s detection performance on edge devices with limited computational power (see Equations 11–14). Consequently, we proposed the lightweight detection model YOLOC-tiny.

While YOLOC-tiny demonstrated excellent detection performance in tasks involving multi-variety and multiripeness non-green-ripe citrus fruits, several limitations remain. First, as shown in **Figure 9** of the revised manuscript, the model’s detection capability for citrus fruits that are either distant or severely occluded is insufficient. Although these fruits can be detected as the robot moves, detecting small, distant citrus fruits and severely occluded citrus fruits requires further attention. Second, in distinguishing between different citrus varieties and maturities, YOLOC-tiny’s detection accuracy is lower compared to algorithms that detect single-variety, single-maturity fruits (Fu et al., 2019; Apolo-Apolo et al., 2020a). As shown in **Table 5**, the mAP of YOLOC-tiny is slightly lower than that of YOLOv5n in simple environments, although YOLOC-tiny outperforms YOLOv5n in complex orchards and varying lighting conditions.

Moreover, in this study, we only verified the impact of adding pure citrus image datasets on enhancing the detection performance of citrus fruits in unstructured environments, without conducting quantitative and qualitative research. Considering the basic conditions of the robot’s operating environment, we used only seven data augmentation methods. Furthermore, transformers have proven effective in large language models and have recently been applied to object detection tasks, suggesting promising avenues for improving model performance (Zhu et al., 2022; Yang et al., 2023). We also note the recent advancements with YOLOv9 and YOLOv10.

We will further expand the dataset, enrich the images with various scenes and lighting conditions, or increase the image resolution. We will explore the effectiveness of generative adversarial networks and MixUp in robot applications in future research. Therefore, future work will focus on enriching the dataset and incorporating more efficient network architectures and modules to further enhance the model’s detection performance and lightweight characteristics. In our future research, we plan to optimize the model structure further to improve the detection performance of citrus fruits in low-light environments.

To address these issues, we will expand the dataset, enhance image diversity with various scenes and lighting conditions, and increase the image resolution (Wang et al., 2022b). Additionally, we will explore the effectiveness of generative adversarial networks and MixUp in dataset augmentation. Future work will focus on incorporating more efficient network architectures and modules to enhance the model’s detection performance and lightweight characteristics. We also plan to optimize the model structure to improve the detection of citrus fruits in low-light environments.

## 5 Conclusions

We introduce a generalized lightweight detection model, YOLOC-tiny, tailored for large non-green-ripe citrus of different varieties with multiripeness in complex environments by optimizing the network structure and reducing the model size to enhance computational efficiency. Our methodology begins with the curation of image datasets featuring citrus fruits in various environments and ripeness stages, encompassing navel orange, Ehime Jelly orange, and Harumi tangerine. YOLOC-tiny utilizes the EfficientNet-B0 feature extraction backbone, streamlining model parameters whilst augmenting feature extraction capabilities. Furthermore, it integrates a spatial and channel hybrid attention mechanism, CBAM, to enhance access to contextual information, intensify focus on diverse citrus fruits, and achieve superior detection performance. Additional parameter reduction is achieved by implementing the LAMP strategy.

The key findings from our study include the following: (1) Ablation experiments confirm the effectiveness of our enhancement measures in improving network performance for non-green-ripe citrus fruit detection. (2) Compared with TRAIN-A, YOLOv7 based on the F-TRAIN-A dataset exhibits a 2.4% and 0.8% improvement in P and mAP, respectively. This finding validates the benefit of replacing citrus images in real scenes with a small number of pure citrus images in complex environments to enhance model detection performance. (3) Compared with other SOTA models, such as YOLOv8, YOLOC-tiny surpasses real-time detection requirements with an impressive frame rate. It also demonstrates superior detection performance. YOLOC-tiny achieves an 85.3% P and an 83.0% mAP at a frame rate of 80 FPS, with a parametric count of merely 4.2 M. (4) In a real-world deployment with a citrus-picking robot, ICPR, YOLOC-tiny attains 92.8% accuracy at a frame rate of 59. Thus, YOLOC-tiny provides real-time, accurate information on multiripeness and diverse citrus fruits for orchard robots.

## Data availability statement

The original contributions presented in the study are included in the article/**Supplementary Material**. Further inquiries can be directed to the corresponding author.

## Author contributions

ZT: Funding acquisition, Resources, Writing – original draft, Writing – review & editing, Conceptualization, Data curation, Formal analysis, Investigation, Methodology, Project administration, Software, Supervision, Validation, Visualization. LX: Conceptualization, Data curation, Formal analysis, Funding acquisition, Investigation, Methodology, Project administration, Resources, Supervision, Writing – review & editing. HL: Data curation, Formal analysis, Software, Validation, Visualization, Writing – review & editing. MC: Conceptualization, Data curation, Funding acquisition, Supervision, Validation, Writing – review & editing. XS: Conceptualization, Data curation, Formal analysis, Investigation, Methodology, Software, Validation, Writing – review & editing. LZ: Data curation, Validation, Writing – review & editing, Methodology, Software. YW: Conceptualization, Data curation, Funding acquisition, Writing – review & editing. ZW: Funding acquisition, Methodology, Resources, Writing – review & editing. YZ: Data curation, Formal analysis, Resources, Writing – review & editing. KR: Software, Validation, Writing – review & editing. YH: Funding acquisition, Resources, Writing – review & editing. WM: Funding acquisition, Resources, Writing – review & editing. NY: Funding acquisition, Resources, Writing – review & editing. LL: Funding acquisition, Resources, Writing – review & editing. YQ: Funding acquisition, Supervision, Writing – review & editing.

## Supplementary material

Supplementary Figure 1 The MBConv convolutional network.

Supplementary Figure 2 Diagram of LAMP for PL-YOLO-tiny.

Supplementary Table 1 Details of citrus fruit image datasets.

Supplementary Table 2 Overview of the dataset and the number of images used in this study.
