# Supplementary figures and images for: YOLOC-tiny: a generalized lightweight real-time detection model for multiripeness fruits of large non-green-ripe citrus in unstructured environments
Source: Front Plant Sci. 2024 Jul 5;15:1415006. doi: 10.3389/fpls.2024.1415006 (PMC11257917; doi:10.3389/fpls.2024.1415006)

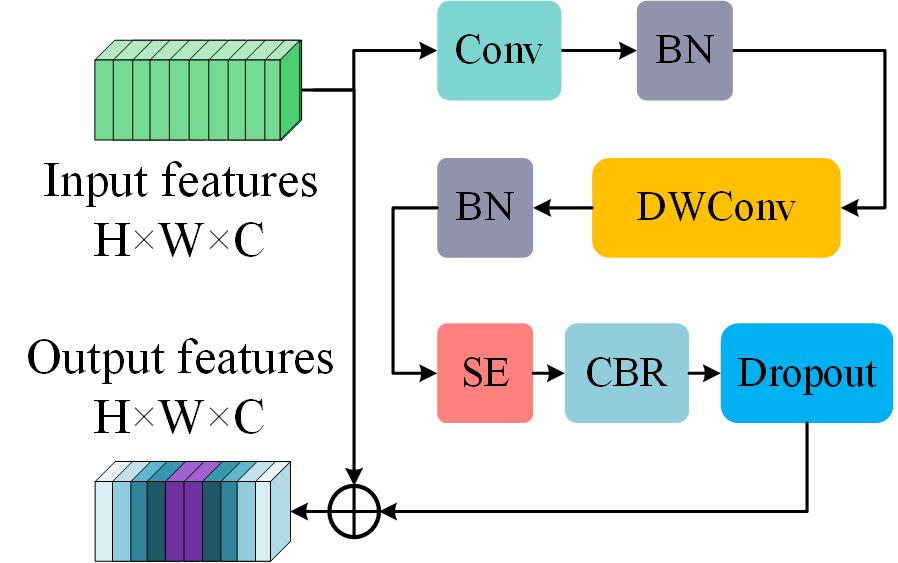

Supplement: Supplementary Figure 1 — The MBConv convolutional network. [file Image_1.jpg]

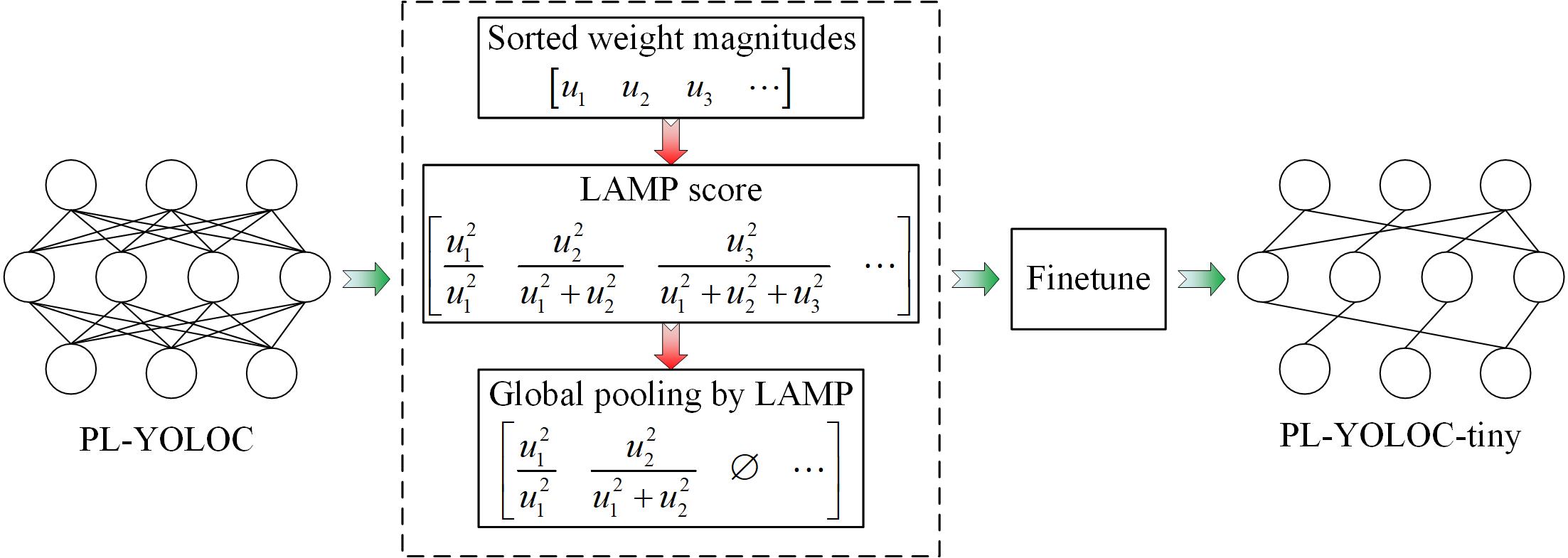

Supplement: Supplementary Figure 2 — Diagram of LAMP for PL-YOLO-tiny. [file Image_2.jpg]
